# Phenotypic and genome-wide association analyses for nitrogen use efficiency related traits in maize (*Zea mays* L.) exotic introgression lines

**DOI:** 10.3389/fpls.2023.1270166

**Published:** 2023-10-09

**Authors:** Darlene L. Sanchez, Alice Silva Santana, Palloma Indiara Caproni Morais, Edicarlos Peterlini, Gerald De La Fuente, Michael J. Castellano, Michael Blanco, Thomas Lübberstedt

**Affiliations:** ^1^Department of Agronomy, Iowa State University, Ames, IA, United States; ^2^Department of Agronomy, State University of Maringá, Maringá, PR, Brazil; ^3^Department of Agriculture, Agricultural Research Service (USDA-ARS), Ames, IA, United States

**Keywords:** candidate gene, quantitative trait locus, diversity, genetic resources, abiotic stress

## Abstract

Nitrogen (N) limits crop production, yet more than half of N fertilizer inputs are lost to the environment. Developing maize hybrids with improved N use efficiency can help minimize N losses and in turn reduce adverse ecological, economical, and health consequences. This study aimed to identify single nucleotide polymorphisms (SNPs) associated with agronomic traits (plant height, grain yield, and anthesis to silking interval) under high and low N conditions. A genome-wide association study (GWAS) was conducted using 181 doubled haploid (DH) lines derived from crosses between landraces from the Germplasm Enhancement of Maize (BGEM lines) project and two inbreds, PHB47 and PHZ51. These DH lines were genotyped using 62,077 SNP markers. The same lines from the *per se* trials were used as parental lines for the testcross field trials. Plant height, anthesis to silking interval, and grain yield were collected from high and low N conditions in three environments for both *per se* and testcross trials. We used three GWAS models, namely, general linear model (GLM), mixed linear model (MLM), and Fixed and Random model Circulating Probability Unification (FarmCPU) model. We observed significant genetic variation among the DH lines and their derived testcrosses. Interestingly, some testcrosses of exotic introgression lines were superior under high and low N conditions compared to the check hybrid, PHB47/PHZ51. We detected multiple SNPs associated with agronomic traits under high and low N, some of which co-localized with gene models associated with stress response and N metabolism. The BGEM panel is, thus, a promising source of allelic diversity for genes controlling agronomic traits under different N conditions.

## 1 Introduction

Nitrogen (N) is critical to promote crop growth and development and to increase grain yield. In cereals such as maize, the application of N fertilizers is an essential agronomic practice (Nag and Das, 2022). Although N fertilizer markedly improves the yield of maize, its excessive use often leads to run-off, which causes the eutrophication of rivers and other bodies of water (Wani et al., 2021). In this context, more than half of the N fertilizer applied to maize is lost to the environment (Ladha et al., 2016; Yu et al., 2022). As an example, N leaching from maize-based cropping systems is the primary cause of hypoxia in the Gulf of Mexico (Goolsby et al., 2000; Alexander et al., 2007). Hence, it is increasingly important to screen genotypes for N use efficiency (NUE) and explore those that have higher NUE and are better suited to N limitation.

Improving NUE in maize would not only help to reduce N fertilization in the field but may also increase productivity in N-deficient environments. However, NUE is a complex trait in which interactions between genetic and environmental factors are involved. Traits such as anthesis-silking interval, plant height, and grain yield have the potential to be used as parameters for NUE screening, since they play an essential role in N acquisition and N utilization in maize, the two main components of NUE (Gheith et al., 2022). NUE-related traits have been successfully used in maize (Kumari et al., 2021), rice Liu et al. (2016b), and potatoes (Getahun et al., 2020) to identify genotypes with better performance under low N conditions. In addition, studies combining quantitative genetics and molecular markers support a strategy of great potential for plant breeders to analyze the genetic architecture of complex traits such those related to NUE. In this context, genome-wide association studies (GWAS) have been widely used to capture complex trait variation down to the genome level by exploring both historical and evolutionary recombination events in maize (Verzegnazzi et al., 2021; Ma et al., 2022; Wu et al., 2022; Xu et al., 2023).

In US elite germplasm, only a small fraction of the total available genetic diversity in maize (<10 out of 300 maize races) is currently used (Andorf et al., 2019). The Germplasm Enhancement in Maize (GEM) project of United States Department of Agriculture—Agricultural Research Service (USDA-ARS) has the objective of improving maize productivity by broadening the genetic base of commercial maize cultivars through evaluating, identifying, and introducing useful genes from maize landraces (Pollak, 2003; Salhuana and Pollak, 2006). In the allelic diversity component of the GEM project, doubled haploid (DH) lines were derived from BC1F1 or F1 crosses between tropical and subtropical accessions and elite inbreds PHB47 (stiff stalk) and PHZ51 (non-stiff stalk), which are expired plant variety protection (ex-PVP) lines (Brenner et al., 2012), to enable photoperiod adaptation of these materials to Midwest US conditions. Currently, the released DH lines are known as BGEM lines, where B indicates Iowa State University, the place where the DH lines were developed (Vanous et al., 2018).

In this study, BGEM lines *per se*, and their testcrosses, were evaluated in field trials under low and high (normal) N conditions for agronomic traits related to NUE. GWAS analyses for the agronomic traits under low (LN) and high N (HN) conditions were conducted. The main objective was to identify novel alleles associated with agronomic traits under low N conditions, which can aid in improving NUE in maize. The specific objectives were to (i) determine the extent of variation of agronomic traits for the BGEM panel grown under HN and LN conditions, (ii) establish correlations among the agronomic traits, (iii) identify associations between SNP markers and agronomic traits grown under HN and LN conditions, and (iv) evaluate the co-localization of these SNPs with putative candidate genes and/or previously identified QTL for traits related to NUE in the inbred and testcross populations.

## 2 Materials and methods

### 2.1 Plant materials

In total, 66 GEM accessions from Central and South America were crossed with the expired PVP lines PHB47 and PHZ51. Most of the F_1_ seeds were backcrossed once with PHB47 and PHZ51, respectively, to produce the BC_1_F_1_ generation as described in Sanchez et al. (2018). A total of 181 BGEM lines and inbred lines PHB47 and PHZ51 were used in *per se* field trials. The DH lines were produced using the protocol described by Vanous et al. (2017), wherein BC_1_F_1_ or F_1_-derived crosses between GEM accessions and PHB47 or PHZ51 were crossed with the inducer hybrid RWS 9 × RWK-76 (Röber et al., 2005) to produce haploid seed, which was identified based on the *R-nj* color marker Liu et al. (2016a). In the subsequent planting season, putative haploids were grown in the greenhouse, where colchicine treatment was applied to seedlings at the three to four leaf developmental stage to promote genome doubling. Haploid plants were transplanted in the field and self-pollinated to produce DH lines. Seed of these lines was increased at the USDA North-Central Region Plant Introduction Station in Ames, Iowa during the summer of 2013 and at the Iowa State University Agricultural Engineering and Agronomy Farm in 2014. In total, 74 and 105 DH lines were obtained from the crosses with the recurrent parents PHZ51 (non-stiff stalk) and PHB47 (stiff stalk), respectively.

The same lines from the *per se* trials were used as parental lines for the testcross field trials. They were divided according to heterotic group membership (i.e., stiff-stalk and non-stiff stalk), and each group was planted in separate isolation plots in Ames during the summer of 2014. Two rows and two ranges of pollen parent surrounded each isolation plot. Inside, for every two rows of female, there was one row of male. There were three replications or rows of each DH line, randomly distributed per isolation plot. In one isolation plot, all lines belonging to the stiff-stalk group (e.g., DH lines with PHB47 as recurrent parent) that were used as female parents were detasseled before anthesis, and PHZ51 was used as pollen parent. In the other isolation plot, all non-stiff stalk lines (e.g., DH lines with PHZ51 as recurrent parent) were detasseled and crossed with PHB47. In total, 74 and 105 testcrosses obtained from the cross with PHZ51 and PHB47, respectively, were evaluated.

### 2.2 Field trials

In this study, a combination of location and year was considered as an environment. Within each environment, two N conditions were evaluated: HN and LN. No fertilizer was applied within the LN condition in all environments. For the HN condition, 261.60 kg N ha^−1^ was applied in the form of 32% urea–ammonium nitrate (UAN) fertilizer before planting via liquid broadcast and immediately incorporated with tillage. Three environments were used for the *per se* trials: at Iowa State University Agricultural Engineering and Agronomy Farm (42.0204° latitude, −93.7738° longitude, 335 m elevation) in Ames, IA, during the summers of 2014 (Ames 2014) and 2015 (Ames 2015), and at the Iowa State University Northeast Research and Demonstration Farm (42.93811° latitude, −92.57018° longitude, 317.742 m elevation) in Nashua, IA, during the summer of 2015 (Nashua 2015).

Two environments were used for the testcross trials, which were performed at the same farms from Ames and Nashua during the summer of 2015. No N fertilizer was applied to the Nashua LN location in 2014, and oats were planted in that area before, in order to deplete the soil N content. For the testcross evaluation in Ames 2015, two LN locations were used. One has historically been planted with maize, and no fertilizer has been applied in that location for several years. The other LN location in Ames 2015 did not receive any fertilizer treatment and was planted with non-nodulating soybeans in the previous year (2014). Therefore, for the testcrosses trials, the maize–maize location was referred to as Ames 2015A, and the soybean–maize location was referred to as Ames 2015B. Only one HN location was used for testcrosses trials in Ames 2015 environment.

Soil samples were collected right before sowing, and the samples were analyzed in the Ames trial plots in 2015. Using a probe, 10 samples per location were collected in the top 30 cm of the soil at randomly selected areas, and samples for each trial were bulked, thoroughly mixed, and submitted to the ISU Soil and Plant Analysis Laboratory at the Department of Agronomy to determine total N and carbon (C) content (McGeehan and Naylor, 1988). The results of samples were collected and analyzed in the Ames trial plots in 2015 (**Table 1**). Results reported C and N as the percentage (%) of C or N in the dried sample (g C or N per 100 g sample). For logistical issues, it was not possible to collect soil samples in Nashua.

**Table 1:** Results of soil samples collected and analyzed in the Ames trial plots.

Condition	Trial	N (%)	C (%)	Location
**High N**	*Per se*	0.39	6.30	Ames
	Testcross	0.35	4.10	Ames
**Low N**	*Per se*	0.16	2.02	Ames
	Testcross	0.17	2.09	Ames 2015A
	Testcross	0.17	2.00	Ames 2015B

All trials were planted following a randomized complete block design (RCBD), in two-row plots. Two ranges of filler were planted at the front and back and four rows at the left and right sides of each trial. Each row was 5.64 m long, and the rows were spaced 0.76 m apart. Planting density was 65,323 plants ha^−1^.

### 2.3 Agronomic traits evaluated

Plant height (PHT) and grain yield (GY) were measured in all trials, while anthesis to silking interval (ASI) data were only collected at the Ames trials. ASI was calculated using the difference in growing degree units (GDUs) between anthesis and silking times. Days to anthesis was recorded as the number of days from sowing to the day when 50% of the plants in the plot had anthers extruded outside the glumes. Days to silking were recorded as the number of days from sowing to the day when 50% of the plants in the plot had silks emerging from the ears. Days to anthesis and silking were converted to growing degree units (GDUs), which were calculated according to the following equation: $GDUs=\frac{T_{max}+T_{min}}{2}$ , where *T_max_* is the maximum daily temperature which is set to 30°C when *T_max_* exceed 30°C, and *T_min_* is the minimum temperature and is set to 10°C when *T_min_* falls below 10°C. PHT in centimeters was taken from the ground surface to the topmost end of the central tassel spike. GY was obtained from two-row plots using a harvesting combine, where grain weight and moisture content were measured. Yield in tons per hectare was computed after moisture content was adjusted to 15.50%.

### 2.4 Statistical analysis of agronomic traits

Data analysis was performed separately for the *per se* and testcross trials fitting the following linear model: $Y_{ijkl}=\mu +E_{i}+R{\left(E\right)}_{ij}+N_{l}+EN_{il}+G_{k}+EG_{ik}+NG_{lk}+ENG_{ikl}+\varepsilon_{ijkl}$ , where *Y_ijkl_* is the observation in the *k^th^* genotype in the *j^th^* replication in the *i^th^* environment and *l^th^* N rate; *μ* is the overall mean; *E_i_* is the effect of the *i^th^* environment; *R*(*E*)*_ij_* is the effect of *j^th^* replication nested within the *i^th^* environment; *N_l_* is the effect of the *l^th^* N rate; *EN_il_* is the interaction effect of the *i^th^* environment and *l^th^* N rate; *G_k_* is the effect of the *k^th^* genotype; *EG_ik_* is the effect of the interaction of the *i^th^* environment with the *k^th^* genotype; *EG_lk_* is the effect of the interaction of the *l^th^* N rate with the *k^th^* genotype; *ENG_ikl_* is the effect of the interaction of the *i^th^* environment and *l^th^* N rate with the *k^th^* genotype; and *ε_ijkl_* is the residual error.

The procedure PROC MIXED from the software package SAS (SAS Institute Inc., North Carolina, USA) was used to perform the analysis of mixed model, where N rate was fixed, and the other factors were random. Variance components, $\sigma_{g}^{2},\sigma_{g\times e}^{2},\sigma_{e}^{2}$ , were estimated accordingly, where $\sigma_{g}^{2},\sigma_{g\times e}^{2},\sigma_{e}^{2}$ correspond to the genotypic variance, genotype by environment interaction variance, and error variance, respectively. Broad-sense heritability (*h*^2^) on an entry mean basis for each trait under each N condition and in the combined analysis were estimated as follows (Hallauer et al., 2010): $h^{2}=\frac{\sigma_{\text{g}}^{2}}{\sigma_{\text{g}}^{2}+\frac{\sigma_{e}^{2}}{\text{r}}}$ and $h^{2}=\frac{\sigma_{g}^{2}}{\sigma_{g}^{2}+\frac{\sigma_{g}^{2}\times e}{n}+\frac{\sigma_{e}^{2}}{rn}}$ , where r is the number of replications within each environment, and n is the number of environments.

For each N condition, best linear unbiased predictions (BLUPs) from all inbred lines and testcrosses across the environments were estimated for all measurements. This was also implemented using PROC MIXED in SAS 9.3 (SAS Institute Inc., 2011). The BLUPs from the combined analysis within each N condition were used to calculate Pearson correlations among traits using PROC CORR function in SAS 9.3 (SAS Institute Inc., 2011).

### 2.5 Molecular marker data

The BGEM lines were genotyped using 955,690 genotyping-by-sequencing (GBS) markers (Elshire et al., 2011). GBS data were generated at the Cornell Institute for Genomic Diversity (IGD) laboratory. After filtering out markers with more than 25% missing data, below 2.5% minor allele frequency, and monomorphic markers, 247,775 markers were left for further analyses. For markers at the same genetic position (0 cM distance), only one marker was randomly selected. The final number of markers used for further analyses was 62,077 markers distributed across all 10 chromosomes.

The average number of recombination events per line was substantially greater than expected. Therefore, the genotypic data were corrected for monomorphic markers that were located between flanking markers displaying donor parent genotypes. The correction was based on Bayes theorem, with an underlying assumption that very short distances of a marker with recurrent parent (RP) genotype to flanking markers with donor genotype are more likely due to identity of marker alleles for that particular SNP between RP and donor, instead of a rare double recombination event. These short RP segments interspersed within donor segments were tested for the null hypothesis that a double recombination occurred and were either corrected or kept as original genotype, accordingly, based on p-values from the Bayes theorem (Vanous et al., 2018). After correction, the donor genome composition was closer to the expected 25%, compared to the original marker data, and the average number of recombination events was substantially reduced (Sanchez et al., 2018). Genotype data of the testcrosses were generated using the “create hybrid genotypes” function in TASSEL 5.2.61 (Bradbury et al., 2007) with genotype information from the BGEM lines *per se*, PHB47 and PHZ51.

### 2.6 Genome-wide association studies

BLUPs from the combined analysis of the traits ASI, PHT, and GY for HN and LN conditions, in the *per se* and testcross trials, were used for GWAS. In order to balance false-positives and false-negatives in detecting significantly associated SNPs, three statistical models were implemented, namely, (1) General Linear Model (GLM) + PCA (Q), where the PCA output from GAPIT was used as a covariate to account for fixed effects due to population structure; (2) Mixed Linear Model (MLM; Yu et al., 2006), where PCA and kinship (K) were used as covariates; and (3) FarmCPU (Fixed and random model Circulating Probability Unification), where Q was also used as covariate, but has additional algorithms to solve the confounding problems between testing markers and covariates Liu X. et al. (2016). The R package GAPIT (Lipka et al., 2012) was used to conduct GWAS for all three models. Additive genetic model was implemented when performing GWAS for *per se* trials, while dominant genetic model was used for the testcross trials.

Multiple testing in GWAS was accounted for using the statistical program simpleM (Gao et al., 2010; Johnson et al., 2010), which calculates the number of informative SNPs (Meff_G) using R statistical software (R Core Team, 2014). First, a correlation matrix for all markers was constructed, and the corresponding eigenvalues for each SNP locus were calculated. GAPIT (Lipka et al., 2012) was then used to calculate a composite linkage disequilibrium (CLD) correlation directly from the SNP genotypes, and once this SNP matrix was obtained, *Meff_G* was calculated, and this value was used to compute for the multiple testing threshold in the same way as the Bonferroni correction method, where the significance threshold (α=0.05) was divided by the *M_eff_G_* (*α*/*M_eff_G_*). For this study, based on the α level of 0.05, the multiple testing threshold level was set at 8.10 × 10^−7^.

The available maize genome sequence (B73; RefGen_v4) was used as the reference genome for candidate gene identification. Candidate genes were identified using the Ensembl Biomart tool (Kinsella et al., 2011). Genes were considered as candidates if a significantly associated SNP marker with phenotypic variance explained (PVE) higher than 10% was located within the range of linkage disequilibrium (LD) decay observed for each chromosome (upstream and downstream). Candidate genes corresponding to each SNP were checked according to the SNP marker’s physical position in the MaizeGDB molecular marker database (http://www.maizegdb.org; Portwood et al., 2019). Functional annotations of candidate genes were predicted in NCBI (http://www.ncbi.nlm.nih.gov/gene) and were also compared to previously published candidate genes.

## 3 Results

### 3.1 Field performance of BGEM lines *per se* under high and low nitrogen conditions

According to the soil chemical analysis (**Table 1**), the N content at LN trials was considerably lower than at HN trials, indicating that the N-depleting effort had been successful in reducing N levels. In addition, all measured traits were affected by N conditions, and most of them had their means reduced by the N deficiency (**Table 2**). We observed wide ranges on the tested traits under LN and HN (**Table 2**). However, the N stress negatively affected the genotypic variation among the DH lines, and the ranges were much larger under HN than under LN for almost all traits, except for ASI in Ames 2014. For this trait, the range under LN was equal to 104.98, while under HN, it was equal to 92.67. On the other hand, traits such as PHT presented wider ranges under HN conditions. In Ames 2014, PHT ranged from 158.60 cm to 272.04 cm under HN and from 159.90 cm to 240.33 cm under LN. In Ames 2015, the same trait had a ranged from 181.96 cm to 289.74 cm under HN and from 141.66 cm to 215.00 cm under LN. In general, higher values of standard deviation (SD) were also observed under HN conditions. For example, ASI had SD equal to 18.36 under HN in Ames 2015, while under LN, it was equal to 12.34. In Ames 2014, PHT had SD equal to 20.36 under HN and 16.25 under LN.

**Table 2:** Summary statistics of agronomic traits in BGEM lines *per se* and testcrosses grown under different N conditions.

Environment	Trait	Low N				High N				Mixed models analysis		
		Mean	Max	Min	H^2^	Mean	Max	Min	H^2^	${\hat{\sigma }}_{G}^{2}$	${\hat{\Phi }}_{N}$	${\hat{\sigma }}_{NG}^{2}$
BGEM lines *per se*												
Ames 2014	ASI	25.00	85.45	−19.53	0.57	10.74	67.59	−25.08	0.51	1,237.76**	95,296.22**	232.06 *
	PHT	197.27	240.33	159.90	0.75	222.04	272.04	158.60	0.82	379.13**	128,974.98**	5.25*
	GY	2.18	3.92	0.91	0.29	2.92	5.85	0.66	0.81	0.76**	75.41**	0.26**
Ames 2015	ASI	37.85	83.06	11.73	0.28	19.62	106.78	14.84	0.61	1,365.54**	209,157.88**	40.97ns
	PHT	180.59	215.00	141.66	0.58	226.85	289.74	181.96	0.63	368.49**	397,964.28**	22.29ns
	GY	1.03	2.05	0.71	0.24	2.37	4.68	1.18	0.42	0.29**	246.94**	0.25**
Nashua 2015	PHT	228.38	276.13	168.60	0.78	240.41	296.12	182.96	0.83	22.14**	27,976.98**	7.84ns
	GY	3.01	5.13	1.21	0.66	4.21	6.96	1.38	0.69	1.30**	182.83**	0.32**
Combined	ASI	31.44	99.34	−4.68	0.42	15.21	95.69	−16.97	0.30	35.50**	270,250.54**	10.27**
	PHT	202.10	245.65	159.56	0.61	229.78	281.77	177.27	0.59	402.81**	519,339.69**	0.43ns
	GY	2.10	3.44	1.19	0.21	3.17	5.55	0.97	0.40	0.61**	319.50**	0.22**
Testcrosses												
Ames 2015A	ASI	19.91	43.29	10.05	0.24	−0.14	23.29	−11.25	0.04	238.23**	25,7926.92**	23.30ns
	PHT	248.75	278.38	207.56	0.41	334.44	379.48	284.45	0.68	232.32**	8,763.92**	41.46*
	GY	3.48	4.68	2.35	0.30	8.27	12.00	3.98	0.62	0.44**	1,890.28**	1.05**
Ames 2015B	ASI	19.84	35.89	12.67	0.25	–	–	–	–	149.44**	228,403.25**	53.88**
	PHT	259.71	285.67	220.41	0.60	–	–	–	–	246.01**	52,119.82**	24.95*
	GY	4.35	5.95	2.32	0.56	–	–	–	–	0.97**	1,559.56**	0.68**
Nashua 2015	PHT	297.44	322.20	248.66	0.72	318.69	346.89	266.96	0.58	202.87**	105,567.88**	138.23ns
	GY	8.29	8.86	7.72	0.09	11.11	16.11	6.39	0.37	0.73**	3,535.22**	0.46**
Combined	ASI	19.87	46.20	8.58	0.23	−0.14	23.17	−11.19	0.53	125.32**	231,387.94**	114.81**
	PHT	268.64	296.37	217.35	0.49	326.57	367.56	269.18	0.69	219.66ns	39,175.14**	4.23ns
	GY	5.37	6.37	3.95	0.28	8.28	11.76	4.93	0.52	0.74**	3,427.98**	0.83ns

a ASI, anthesis to silking interval (GDU); PHT, plant height (cm); GY, Grain Yield (t ha^−1^), H^2^, broad-sense heritability; ${\hat{\sigma }}_{G}^{2}$ , genotypic variance component estimate; ${\hat{\Phi }}_{N}$ , quadratic component of nitrogen fixed effect; ${\hat{\sigma }}_{NG}^{2}$ , variance component of nitrogen rate by genotype interaction; *significant at p = 0.05; **significant at p = 0.01; ns, not significant.(-) means data were not collected.

PHT and GY were affected by N deficiency and had their means reduced under LN conditions (**Table 2**). While the mean of PHT under HN was equal to 222.04, it was equal to 197.27 cm under LN condition in Ames 2014. In Ames 2015, GY had a mean of 2.37 t ha^−1^ under HN, while under LN, it was equal to 1.03 t ha^−1^. On the other hand, ASI had higher means under LN than under HN condition. In Ames 2014 and Ames 2015, ASI had means equal to 10.74 and 19.62 under HN, respectively, and equal to 25.00 and 37.85 under LN, respectively. We observed that GY was the trait most negatively affected by N deficiency and presented the highest mean reduction in response to the LN across all environments. The decrease in the mean under LN compared to HN was equal to 25.34%, 56.54%, 28.50%, and 33.75% in Ames 2014, Ames 2015, and Nashua 2015 and in the combined analysis, respectively.

Variance components due to genotype were highly significant (*p*< 0.01) by the likelihood ratio test for all traits in the *per se* trials (**Table 2**). In addition, variance components due to genotypes × N rates interaction were highly significant (p< 0.05) for almost all traits. In general, the heritability estimates were higher under HN than under LN conditions. For example, GY heritability estimate under HN in Ames 2014 was equal to 0.81, while under LN condition, it was equal to 0.29. In Ames 2015, ASI had heritability equal to 0.61 under HN and equal to 0.28 under LN condition. In the combined analysis, the heritability estimates were low to intermediate (<0.70). In this context, GY had the lowest heritability estimate under LN condition (0.21) and the intermediate one under HN (0.40). Across all environments, the highest yielding BGEM lines under LN were BGEM-0137-S, BGEM-0044-S, BGEM-0127-N, and BGEM-0243-S with GY ranging from 3.12 t ha^−1^ to 3.44 t ha^−1^, and 52 out of the 179 BGEM lines performed better than PHB47 (GY = 2.41 t ha^−1^). On the other extreme, DH lines BGEM-0223-N, BGEM-0225-N, BGEM-0247-N, BGEM-0237-N, and BGEM-0165-S performed poorly with yields ranging from 1.19 t ha^−1^ to 1.30 t ha^−1^.

### 3.2 Performance of testcrosses under high and low nitrogen conditions

Similar to the *per se* trials, the ranges were much larger under HN than under LN for almost all traits, except for ASI in the combined analysis (**Table 2**). This difference was even more pronounced with GY. In Ames 2015A, GY values ranged from 3.98 t ha^−1^ to 12.00 t ha^−1^ under HN, while under LN, it ranged from 2.35 t ha^−1^ to 4.68 t ha^−1^. In Nashua 2015, GY ranged from 6.39 t ha^−1^ to 16.11 t ha^−1^ under HN and from 7.72 t ha^−1^ to 8.86 t ha^−1^ under LN condition. In general, SD values were also higher under HN conditions, except for ASI in Ames 2015A and in the combined analysis. For GY in Ames 2015A, the SD was equal to 1.43 and 0.39 under HN and LN conditions, respectively. PHT and GY were affected by N conditions, and their means reduced with the N deficiency. The percentage of reduction in the mean was stronger for GY. The GY reduction mean was equal to 57.92%, 25.38%, and 35.14% in Ames 2015A, Nashua 2015, and in the combined analysis, respectively (**Table 2**). Conversely, ASI increased its means under LN condition. In Ames 2015A, ASI means were equal to −0.14 and 19.91 under HN and LN conditions, respectively.

The statistical analysis conducted within environment for testcrosses showed that, for almost all traits, there was significant effect of genotype (*p<* 0.01), except for PHT in the combined analysis. Variance components due to genotypes × N rates interaction were highly significant (*p*< 0.01) for GY in all environments, while for ASI and PHT, the significance depended on the environment where they were evaluated. In relation to the heritability estimates within environments, we observed that PHT had the highest estimates among the three traits, ranging from 0.41 to 0.72 under LN and from 0.58 to 0.68 under HN. The heritability estimates for GY ranged from 0.09 to 0.56 under LN and from 0.37 to 0.62 under HN (**Table 2**). In general, heritability estimates in the testcross trials across environments were higher under HN than under LN. For example, in the combined analysis of ASI, heritability estimates under HN were equal to 0.53 and 0.23 under LN.

Testcrosses performing best under LN across environments were BGEM-0258-S/PHZ51, BGEM-0112-S/PHZ51, BGEM-0070-S/PHZ51, BGEM-0115-S/PHZ51, BGEM-0233-S/PHZ51, and BGEM-235-N/PHB47, with yields ranging from 6.13 t ha^−1^ to 6.33 t ha^−1^. The lowest yields were obtained for BGEM-0166-S/PHZ51, BGEM-0263-S/PHZ51, BGEM-0269-S/PHZ51, BGEM-0078-S/PHZ51, and BGEM-00129-N/PHB47, ranging from 3.95 t ha^−1^ to 4.19 t ha^−1^. GY of the checks, PHB47/PHZ51 and its reciprocal PHZ51/PHB47, under LN were 6.37 t ha^−1^ and 5.85 t ha^−1^, respectively. Testcrosses outperforming the GY of PHB47/PHZ51 were identified in the Ames environments. In Ames 2015B environment, there were testcrosses that outperformed PHB47/PHZ51, with BGEM-0112-S/PHZ51, BGEM-0155-S/PHZ51, and BGEM-0226-S/PHZ51 performing better than PHB47/PHZ51 under both LN and HN. The testcrosses BGEM-0001-N/PHB47, BGEM-0044-S/PHZ51, BGEM-0111-S/PHZ51, BGEM-0114-S/PHZ51, and BGEM-0115-S/PHZ51 performed consistently better than PHB47/PHZ51 under the two LN environments in Ames.

### 3.3 Correlations among and within *per se* and testcross agronomic traits

Within BGEM lines *per se*, significant and close positive correlations were observed for PHT evaluated under different N conditions (r = 0.91), and GY (r = 0.69) and ASI (r = 0.75; **Table 3**). Moderate negative correlations were observed between ASI and GY under HN (r = −0.50) and LN (r = −0.48). Within the testcross, a high positive correlation was observed between PHT under HN and PHT under LN condition (r = 0.78) and between GY and PHT under LN (r = 0.66). ASI under HN was not significantly correlated with neither GY under HN and LN nor with PHT under LN. There were also no significant correlations observed between ASI under LN and PHT under HN and GY under HN (**Table 3**). In addition, there was no strong correlation (r > 0.60) between GY with the other two traits neither under HN nor under LN for BGEM lines and their testcrosses. Therefore, according to our results, we could not use PHT and ASI as indirect selectors for GY.

**Table 3:** Pearson correlation of agronomic traits in BGEM lines and testcrosses grown under low nitrogen (LN) and high nitrogen (HN) conditions across environments.

Trait		HN			LN		
		GY^a^	PHT	ASI	GY	PHT	ASI
HN	GY		0.36**	−0.50**	0.69**	0.29**	−0.42**
	PHT	0.43**		−0.15*	0.14*	0.91**	−0.04^ns^
	ASI	−0.12^ns^	−0.23**		−0.46**	−0.08^ns^	0.75**
LN	GY	0.48**	0.49**	−0.11^ns^		0.18**	−0.48**
	PHT	0.40**	0.78**	−0.11^ns^	0.66**		0.03^ns^
	ASI	−0.07^ns^	−0.14^ns^	0.43**	−0.27**	−0.21**	

Values above the diagonal are correlations among BGEM lines per se, and values below the diagonal are correlations among testcrosses.

**^a^** GY, Grain Yield (t ha^−1^); PHT, plant height (cm); ASI, anthesis to silking interval (GDU); *significant at p = 0.05; **significant at p = 0.01; ^ns^not significant.

Weak to moderate (r< 0.60) correlation coefficients were observed between the performance of testcross and *per se* genotypes (**Table 4**). The highest correlation coefficients were observed between testcross PHT under HN and *per se* lines PHT under HN (r = 0.52) and LN (r = 0.52). Testcross PHT under LN also correlated well with *per se* PHT under both HN (r = 0.49) and LN (r = 0.52). According to the correlation coefficients, there is no possibility to use any trait from the *per se* performance to predict the performance of testcross hybrids under neither N condition.

**Table 4:** Correlations of agronomic traits between BGEM lines *per se* and testcrosses grown under different Nitrogen (N) conditions across environments.

Per se traits		Testcross traits					
		High N			Low N		
		GY^a^	PHT	ASI	GY	PHT	ASI
High N	GY	0.11^ns^	0.14*	−0.16*	0.17*	0.08^ns^	−0.13^ns^
	PHT	0.14*	0.52**	−0.07^ns^	0.15*	0.49**	0.01^ns^
	ASI	0.00^ns^	−0.05^ns^	0.28**	−0.03^ns^	0.01^ns^	0.21**
Low N	GY	0.03^ns^	0.05^ns^	−0.17*	0.19**	0.03^ns^	−0.22**
	PHT	0.12^ns^	0.52**	−0.05^ns^	0.18**	0.52**	0.00^ns^
	ASI	0.03^ns^	0.06^ns^	0.26**	0.01^ns^	0.13^ns^	0.23**

**^a^** GY, Grain Yield (t ha^−1^); PHT, Plant height (cm); ASI, Anthesis to silking interval (GDU); *significant at p=0.05; **significant at p=0.01; ^ns^not significant.

### 3.4 Genome-wide association studies for agronomic traits in *per se* and testcross trials

**Figure 1:**
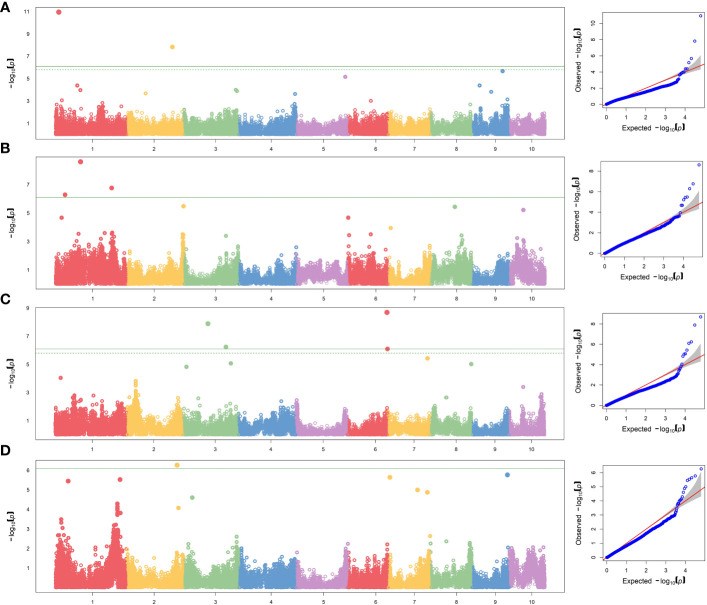
GWAS-derived Manhattan and QQ plots showing significant SNPs associated with **(A)** *per se* anthesis-silking interval under HN, **(B)** testcrosses plant height under LN and **(C)** under HN, and **(D)** testcrosses yield under HN using FarmCPU model. Each dot represents an SNP. The horizontal solid line represents the Bonferroni-corrected significant threshold of 8.10 × 10^−7^.

To reduce the impact of environmental variability, BLUP values across the three environments (Ames 2015A, Ames 2015B and Nashua 2015) were used for association study. No SNPs were found when performing GWAS with MLM model. A total of seven significant SNPs were found by applying FarmCPU and GLM models (**Table 5**; **Figure 1**). The same SNPs detected by FarmCPU were detected by GLM. This result indicates that these common SNPs have high reliability. For simplicity, we presented the results from FarmCPU, and the subsequent analysis mainly focused on those seven SNPs.

**Table 5:** Significant SNP markers information associated with agronomic traits of BGEM lines *per se*, and their testcrosses, grown under high nitrogen (HN) and low nitrogen (LN) conditions.

Trait	SNP	Chr	P-value	Effect	MAF	q-value	PVE (%)
*per se*							
ASI-HN	S1_13685600	1	1.11×10^−11^	7.58	0.18	6.89×10^−7^	12.11
	S2_190189512	2	1.49×10^−8^	−7.36	0.34	4.61×10^−4^	30.18
Testcross							
PHT-LN	S1_39752558	1	5.15×10^−7^	6.70	0.16	0.01	2.53
	S1_104874404	1	2.49×10^−9^	8.80	0.39	1.54×10^−4^	24.81
	S1_235704086	1	1.74×10^−7^	−6.06	0.36	5.40×10^−3^	3.65
PHT-HN	S3_104138066	3	1.30×10^−8^	9.86	0.09	4.03×10^−4^	4.46
	S3_179633217	3	5.75×10^−7^	−8.49	0.13	0.01	19.19
	S6_165585769	6	2.04×10^−9^	10.56	0.14	1.27×10^−4^	1.28
GY-HN	S2_209927372	2	5.44×10^−7^	−0.71	0.09	0.03	40.36

The q-value given is the chromosome-wide FDR-adjusted p-value.

PVE, phenotypic variance explained; GY, Grain yield (t ha^−1^); PHT, plant height (cm); ASI, anthesis to silking interval (GDU).

For the *per se* data, the GWAS analysis identified significant SNPs only for ASI under HN condition. Interestingly, one of the two SNPs (S2_190189512) had PVE >30%. This SNP is within the gene model GRMZM2G414252, located between 190,556,326 and 190,557,054 bp on Chromosome 2. The SNP marker S1_13685600 (P = 1.11×10^-11^, SNP effect = 7.58) was also significantly associated with ASI under HN conditions. The associated gene model GRMZM2G037912 (14,081,196–14,083,562 bp in Chromosome 1) was identified as a putative vesicle-associated membrane protein (**Table 6**).

**Table 6:** Candidate genes associated with agronomic traits of BGEM lines *per se*, and their testcrosses, grown under high nitrogen (HN) and low nitrogen (LN) conditions.

Traits	Chr	Gene start (bp)	Gene ID MaizeGDB	Gene ID Gramene	Gene name	Annotation
*per se*						
ASI - HN	1	13809656	Zm00001d027800	GRMZM2G169280	*ppr*	pentatricopeptide repeat-containing protein
	1	14081196	Zm00001d027808	GRMZM2G037912	*vap726*	putative vesicle-associated membrane protein 726
	1	13863300	Zm00001d027802	GRMZM2G004641	*hb64*	Homeobox-transcription factor 64
	2	190605384	Zm00001d005843	GRMZM2G088242	*hsftf2*	HSF-transcription factor 2
	2	190556326	Zm00001d005841	GRMZM2G414252	*bhlh20*	bHLH-transcription factor 20
	2	190962154	Zm00001d005856	GRMZM2G134502	*nup58*	nucleoporin58
Testcrosses						
PHT - LN	1	105862947	Zm00001d030103	GRMZM2G070271	*umc2230*	probable xyloglucan endotransglucosylase/hydrolase protein 27
	1	105553409	Zm00001d030098	GRMZM2G158976	*vq6*	VQ motif-transcription factor6
PHT - HN	3	177266069	Zm00001d042694	GRMZM2G110897	*poll1*	pollux-like1
	3	177609579	Zm00001d042706	GRMZM2G087619	*pds5a*	sister chromatid cohesion protein PDS5 homolog A
	3	177276266	Zm00001d042695	GRMZM2G110922	*snrkII4*	SnRK2 serine threonine protein kinase 4
	3	177338945	Zm00001d042697	GRMZM2G077333	*psbs1*	photosystem II subunit PsbS1
YLD - HN	2	209512508	Zm00001d006476	GRMZM2G171707	*aco5*	aconitase5
	2	209563817	Zm00001d006479	GRMZM2G168706	*cdpk3*	calcium dependent protein kinase3
	2	209688288	Zm00001d006486	GRMZM2G311187	*prh79*	protein phosphatase homolog79
	2	210172903	Zm00001d006508	GRMZM2G125495	*glr3.4*	glutamate receptor 3.4
	2	209822231	Zm00001d006493	GRMZM2G470075	*mate21*	multidrug and toxic compound extrusion21
	2	210284963	Zm00001d006512	GRMZM2G067063	*pdi12*	protein disulfide isomerase12

For testcross data, three significant SNP markers each were found for PHT under both LN and HN, but did not overlap. None of the SNPs affected more than one trait. The SNP marker S1_104874404 on Chromosome 1 was significantly associated with PHT under LN (P = 2.49×10^−9^, SNP effect = 8.80) with a PVE equal to 24.8%. This SNP is located within the gene model GRMZM2G158976 (105,553,409–105,554,335 bp), and encodes a VQ motif-containing protein. GRMZM2G070271 is 308,612 bp away from GRMZM2G158976 and encodes a xyloglucan endotransglucosylase/hydrolase protein. For PHT under HN conditions, one SNP marker had a PVE higher than 10% (S3_179633217). This SNP marker is within the gene model GRMZM2G087619 (177,609,579–177,634,652 bp), identified as sister chromatid cohesion protein on Chromosome 3. S2_209927372 was significantly associated with GY under HN (*p* = 5.44×10^−7^, SNP effect = −0.71). It is worth noting that this SNP marker explained more than 40% of phenotypic variance. The gene model GRMZM2G311187 (209,688,288–209,689,726) co-locates with this SNP, which encodes for a phosphatase protein. Other putative gene models identified by significant associations are listed in **Table 6**.

## 4 Discussion

### 4.1 Effect of nitrogen deficiency on agronomic traits

Screening maize genotypes for yield-related traits tested under LN conditions and optimal-N conditions is critical for long-term maize production in areas with low N fertility. In our study, we evaluated a panel of BGEM lines and their respective testcrosses. Information about population structure, genetic diversity, and linkage disequilibrium of BGEM lines have been reported (Sanchez et al., 2018; Ma et al., 2020; Zuffo et al., 2022). We observed a significant reduction in GY of BGEM lines and their derived testcrosses when evaluated under LN conditions, confirming the importance of sufficient N supply in maize production. Previous studies reported maize yield losses under N stress ranging from 37% to 78% (Bertin and Gallais, 2000; Presterl et al., 2002; Gallais and Hirel, 2003; Presterl et al., 2003; Abdel-Ghani et al., 2013; Chen et al., 2013; Das et al., 2019). In addition, testcross genotypes had better performance under LN than *per se* genotypes as a consequence of heterosis effect (Hallauer et al., 2010).

N deficiency is an important factor causing low yields in maize. During reproductive stage, N stress induces plant senescence, protein degradation, and thus reduces photosynthesis (Mu and Chen, 2021). To keep high GY in LN conditions, it is crucial to select genotypes with better performance under N stress conditions. Our study identified BGEM lines with outstanding performance under LN conditions. This shows the effectiveness of the DH technique in creating genetic variation that can be exploited in breeding for LN stress tolerance. Furthermore, the high performing lines from the same heterotic group could be used to develop breeding populations, either a synthetic population and/or several biparental populations. These could be used as a germplasm source for the development of new maize inbred lines with high allele frequency for NUE. Conversely, the BGEM lines from opposite heterotic groups might be used as parents in the development of maize hybrids tolerant to N stress conditions.

On average, the increase in ASI due to N deficiency stress was 16.24 GDUs in the per se trials and 20.01 GDUs in the testcross trials. Other studies have also reported an increase in ASI under LN conditions (Lafitte and Edmeades, 1995; Bertin and Gallais, 2000; Presterl et al., 2002; Gallais and Hirel, 2003; Abdel-Ghani et al., 2013; Das et al., 2019). According to Lin and Tsay (2017), the flowering time is postponed by either extreme deficiency or excess of N, while intermediate N concentrations promote flowering. Conversely, PHT means were lower under LN conditions for both *per se* and testcross trials. PHT reduction due to N deficiency stress was also observed in both inbred lines *per se* and testcrosses by Presterl et al. (2002). N is the most limiting nutrient and its rate of application influences maize growth and development at different stages. According to Singh et al. (2022), maize plants grown under LN conditions exhibited visual symptoms of N deficiency such as stunted growth and a significant reduction in shoot biomass. This indicates stress-related growth retardation, highlighting the prominent role of N for biomass accumulation (Qi and Pan, 2022).

Broad sense heritability in LN condition decreased from 0.02 (PHT in the combined analysis) to 0.52 (GY in Ames 2014) in the *per se* trials, and from 0.20 (PHT in the combined analysis) to 0.32 (GY in Ames 2015A) in the testcross trials. Decrease in heritability under stress conditions was also observed in previous studies in both maize inbred lines *per se* (Agrama et al., 1999; Bertin and Gallais, 2000; Gallais and Coque, 2005) and testcrosses (Bänziger et al., 1997; Presterl et al., 2002). Reasons for the decrease in heritability estimates include the decrease in genotypic variances instead of increased error variances (Bänziger et al., 1997; Gallais and Coque, 2005) and higher genotypes by environments interaction under LN than under HN (Gallais and Coque, 2005). The significant genotype × N condition interactions for most of the traits suggests that the genotypes responded differently to the N conditions. According to Presterl et al. (2003), the high variance in the genotype × N interactions emphasizes the need for multi-environment testing to identify N-use efficient cultivars with a broad adaptation to different N levels.

### 4.2 Correlations between *per se* and testcross agronomic traits

Indirect selection for GY based on secondary traits is a cheaper approach compared to direct selection for GY due to relatively high heritability of secondary traits and high genetic correlation between secondary traits and GY under LN conditions. As heritability estimates for GY were low to moderate in our study, significant and close correlations between GY and traits with higher heritability, such as PHT and ASI, would be useful for indirect selection. Moreover, correlations between GY under HN and LN would be useful to predict GY under LN based on HN trials. However, the efficiency of indirect selection depends on the strength of the genetic correlation between the environments or traits. In this context, despite positive correlation between HN and LN conditions for GY, the magnitude of the correlation coefficients in our study was small and non-significant in most cases. While in the *per se* trials, the GY correlation between HN and LN was close to 0.70, the correlation was<0.50 in the testcross trials. This reveals how critical it is to evaluate genotypes under the target environment, for both stress and optimal N conditions. Indirect selection for GY under LN through performances obtained from HN conditions was found to be inefficient in a study conducted by Ertiro et al. (2020). According to the authors, low efficiency of indirect selection was explained by the low correlation between environments that resulted from a high proportion of genotype × N variance.

In our study, significant and moderately negative correlations were observed between GY and ASI in the *per se* trials, while these were not significant in the testcross trials. Silva et al. (2022) also reported a negative association between ASI and GY. Gallais and Hirel (2003) suggested that ASI may have a role in stress tolerance physiology, wherein having a shorter ASI would translate to that genotype having a better N metabolism efficiency, or increased yield under LN conditions. Correlations between PHT under HN and PHT under LN were higher than 0.70 for both *per se* and testcrosses trials, which indicates a possibility to evaluate PHT under only one N condition.

In terms of correlation between traits in BGEM lines *per se* and testcrosses, weak to non-significant correlations were observed between *per se* and testcross data. Therefore, the prediction of testcross performance based on *per se* information does not seem to be feasible for BGEM materials. This prediction is even more difficult for traits showing high heterotic effect, such as GY. Therefore, while the BGEM *per se* lines are mainly under additive genetic control, their testcrosses have the effect of dominance and, potentially, epistasis effects. According to Mihaljevic et al. (2005), an indirect improvement of testcross based on *per se* performance is economically advantageous, but it is only feasible with a high positive correlation between *per se* and testcross performance.

### 4.3 Significant SNP-trait associations detected by GWAS

The MLM model did not detect significant SNPs. The MLM with PCA and K model includes the kinship matrix in the model and is expected to reduce the false positives that arise from family relatedness (Yu et al., 2006). However, advantages of the MLM model to control false positives disappear for complex traits when they are associated with population structure having extensive genetic divergence. Kaler et al. (2019) reported that MLM model was particularly conservative and did not find any significant markers, while the FarmCPU model performed better with a less conservative approach. We used FarmCPU model, a GWAS approach that included population structure and kinship and additional algorithms that were used to address confounding problems between the markers and covariates Liu X. et al. (2016). This makes FarmCPU a GWAS approach that is intermediate between GLM and MLM in terms of stringency. In this context, the majority of candidate genes found in our study are related to stress tolerance. The *bHLH* (Zm00001d005841) displayed a subset of stress-responsive genes in *Arabidopsis* (Smolen et al., 2002). We also found a nuclear pore complex, *nup*, (Zm00001d005856), which is the main transport channel between cytoplasm and nucleoplasm and plays an important role in stress response. According to Liu et al. (2022), the overexpression of *nup58* in maize significantly promoted both chlorophyll content and activities antioxidant enzymes under drought and salt conditions. In addition, the expression patterns of the VQ genes (Zm00001d030098) have been analyzed in stress response in maize. According to Song et al. (2015), VQ motif-containing proteins play crucial roles in abiotic stress responses in plants. The expression profiles of VQ genes were analyzed in response to LN stress in soybean (Wang et al., 2014). The SnRK2 family members (Zm00001d042695) are plant-specific serine/threonine kinases involved in plant response to abiotic stresses and abscisic-acid-dependent plant development (Kulik et al., 2011). The *cdpk* (Zm00001d006479) is one of the well-known Ca^2+^ sensor protein kinases involved in environmental stress resistance (Asano et al., 2012). Several *cdpks* have been shown to be essential factors in abiotic stress tolerance, positively or negatively regulating stress tolerance by modulating abscisic acid signaling and reducing the accumulation of reactive oxygen species (Asano et al., 2012).

In our study, we found one SNP marker (S2_209927372) with over 40% of PEV. Although the literature reports few cases of total PEV higher than 30% for GY (Ajnone-Marsan et al., 1995; Sibov et al., 2003; Liu et al., 2012), the identification of a major GY-associated QTL is unusual. Fundamentally, a significant SNP can be due to a superior allele with potential to increase GY in elite germplasm. Conversely, a significant SNP can be caused by a yield-reducing allele. The latter option seems likely, given that GEM materials are based on non-adapted exotic introgressions. In addition, we observed that the SNPs found under LN did not overlap those found under HN. This result validates the low correlation observed between environments. Under abiotic stress conditions, the physiological mechanisms involved and genes responsible in control of traits may be different. Plants respond to abiotic stress through a variety of physiological, biochemical, and transcriptional mechanisms Waters et al., 2017. Potentially, the genes exhibited altered levels of expression in response to the LN stress, which confirmed the need to screen and select genotypes for each N condition separately. We also observed negative and positive allelic effects. A positive value of allelic effect indicates that the minor allele was the favorable allele associated with the increase in the target trait, and a negative value indicates that the major allele was the favorable allele associated with the target trait (Ertiro et al., 2020).

Our derived DH lines may be promising materials for further studies on NUE or developing lines with improved NUE. SNPs significantly associated with agronomic traits under LN conditions, which can aid in improving NUE in maize. These SNPs can also be used to select for donor lines or superior breeding lines, after validating these putative SNPs by developing near-isogenic lines for linkage or expression analysis, or through transgenic methods. Our study shows that exotic germplasm from the GEM project are, therefore, useful sources of novel genes to select for yield and other agronomic traits under low N to improve NUE in maize.

## Data availability statement

The original contributions presented in the study are publicly available. This data can be found here: https://doi.org/10.25380/iastate.24009039.v1.

## Author contributions

DS: Data curation, Formal Analysis, Methodology, Writing – original draft, Writing – review & editing. AS: Formal Analysis, Writing – original draft, Writing – review & editing. PM: Writing – original draft, Writing – review & editing. EP: Methodology, Writing – original draft, Writing – review & editing. GD: Supervision, Validation, Writing – review & editing. MC: Funding acquisition, Validation, Writing – review & editing. MB: Funding acquisition, Writing – review & editing. TL: Conceptualization, Funding acquisition, Methodology, Project administration, Supervision, Writing – review & editing.
